# Editorial

**Published:** 1853-10

**Authors:** 


### THE MEDICAL EXAMINER.

### PHILADELPHIA, OCTOBER, 1853.

Since the issue of our last number, the yellow fever has continued to present a few cases, all of which have, we believe, been traceable to the same circumscribed area in the south-eastern part of the city, which we have already alluded to. The general health of the town has been remarkably good, the bills of mortality being less than for corresponding periods of last year; and the fever may be said to be dying out, as the cases are not only diminished in number, but are much less malignant in type, than during the first period of its appearance. The frost, which may now be daily expected, will no doubt extinguish entirely the smouldering embers of the disease. We shall give in our November number, a conclusion of the account of the visitation of the fever, which was published in the September number.

---

### MEDICAL DEPARTMENT OF PENNSYLVANIA COLLEGE.

Professor HENRY S. PATTERSON, who has for many years so ably filled the chair of Materia Medica and Therapeutics in this Institution, has retired from the active performance of the duties of that department, in consequence of enfeebled health, and has been appointed Emeritus Professor.

The vacancy thus created, has been filled by the election of Dr. JOHN B. BIDDLE, of Philadelphia, one of the editors of the Medical Examiner, formerly Professor of Materia Medica and Therapeutics in Franklin Medical College, and Lecturer on the same branches in the Philadelphia Medical Institute.

Dr. Biddle has earned an enviable reputation as an accomplished and able teacher, and it is confidently believed that the interests of the School, and especially of the department of Materia Medica, so happily and brilliantly illustrated by his predecessor, will lose nothing in being committed to his hands.

The numerous friends of Dr. Patterson, we are sure, will join us in our heartfelt wishes that the repose he now seeks from arduous duties, may result in his entire restoration to health and usefulness. S.

